# Design and fabrication of a device for cleaning greenhouse roofs

**DOI:** 10.1016/j.heliyon.2025.e41991

**Published:** 2025-01-16

**Authors:** Ahmed Amin, Xiaochan Wang, Zhao Lianyuan, Yinyan Shi, Ren Xiaoyan, Mahmoud Okasha, Reda Hassanien Emam Hassanien

**Affiliations:** a College of Engineering, Nanjing Agricultural University, Nanjing City, 210032, China; b Agricultural Engineering Research Institute (AEnRI), Agricultural Research Center (ARC), Giza, 12311, Egypt; c Agricultural Engineering Department, Faculty of Agriculture, Cairo University, Egypt

**Keywords:** CFD, Dirt accumulation, Greenhouse, Light intensity, Roof cleaner, Velocity

## Abstract

Dust, algae, mold, lichen, and moss accumulation on the greenhouse cover materials could hinder sun lights and decrease the internal light intensity in greenhouses. Moreover, the traditional way of cleaning greenhouse roofs using ladders is laborious and time-consuming, requires a lot of water, and puts too much pressure on the roof. It could cause damage and risky labor. Consequently, many farmers leave greenhouse roofs dirty, which decreases plant photosynthesis due to the low light intensity. Therefore, this study aimed to design and fabricate a device for cleaning greenhouse roofs. The device parts were a telescopic arm with three concentric and adjustable cylindrical pipes with a rough brush mounted on a frame and connected to a motorized knapsack sprayer. The cleaning part of the 3D model was investigated using Computational Fluid Dynamics (CFD) software. The simulation outcomes informed that the system could supply a uniform nozzle speed. Meanwhile, it could control the water pressure and water flow rate. The analysis of the intensity disorder and the dissipation rate showed positive results for the system and valuable insights for efficient and safe system design. The average transmittances of the greenhouse roof before and after cleaning were 48.7 % and 68.5 %, respectively. Results also showed that the nozzle overlaps increased as the height of the mattress arm increased. There was no interference in all nozzle spray angles, and the first interference was recorded at a nozzle spray angle of 65° and a height of up to 19 cm. The overlap was ranged from 3 cm to 5 cm at a nozzle height of 21.5 cm. The ideal nozzle spacing was 27 cm at a nozzle spray angle of 65°. In conclusion, an efficient greenhouse roof-cleaning device has been developed for small and medium farms. However, further research is needed to enhance its power source, extend its lifespan, and improve its overall efficiency.

## Graphical abstract

**Figure:**
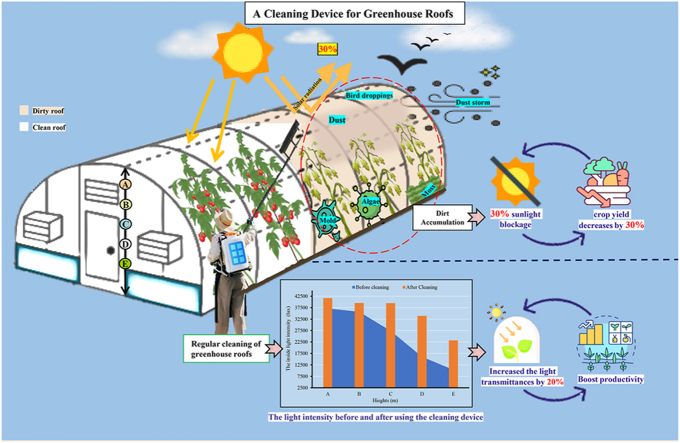

## 1 Introduction

Current demographic projections estimate a population of 9.8 billion by the year 2050. This will cause a considerable 70 % surge in the demand for food, thereby significantly impacting global food security and freshwater resource availability [1,2]. The unequal concentration of the global population in urban centres will heighten existing strains. Besides the challenges posed by population growth and freshwater depletion, global warming and climate change will also affect agricultural production [3,4]. The identified challenges emphasize the importance of agricultural sector investment in ensuring food security and implementing refined resource management policies to reduce climate change's effects on agricultural output.

Greenhouse technology has become a symbol of agricultural innovation, profoundly changing farming practices worldwide. Its impact on agriculture goes beyond borders, as shown by China's experience, which reflects its global significance. By improving off-season vegetable supply and crop output, greenhouse technology has revolutionized agricultural practices in China [5,6]. By 2017, China had assumed a leading global role in climate-smart agriculture, cultivating 41,090 km^2^ of land under greenhouse protection [7].

Various technical methods for crop production have emerged in response to the impending challenge. Greenhouse technology has become a cornerstone of modern agricultural practices [8]. The ability to regulate local environments within greenhouses has proven instrumental in enhancing both the quantity and quality of crop yields per unit area. This shift towards greenhouse is increasingly prevalent on a global scale.

Multi-span greenhouses are now essential parts of protected agriculture industries worldwide, covering vast areas that exceed 1000 km^2^ in China alone [9,10]. However, along with the advantages of greenhouse technology come unique challenges. Over time, dust accumulation on greenhouse surfaces promotes algae growth, compromising light transparency and hindering photosynthesis [11]. This issue is a universal challenge faced by greenhouse operators worldwide. It is essential to keep in mind that dust and condensation can have a negative impact on the light transmission of greenhouses. In fact, a single-cover structure can lose nearly 40 % of its light during winter, and an inflated double-film greenhouse can lose even more. This effect is less significant during the summer months due to less frequent condensation and the angle of incidence being closer to normal. If the dust is high, it can even reduce light transmission below that of glass, negating the advantage of using plastic greenhouses for light-demanding crops [12]. The growth of algae and the darkening of transparent greenhouse materials can reduce light transmittance, thus hindering the growth of crops [13].

Farmers often clean greenhouses manually, but this can be difficult and ineffective due to the size and height of the structures. Researchers have studied cleaning equipment and Chinese market trends. Dust accumulation on greenhouse roofs can reduce photosynthesis and damage structure surfaces [14]. Roofs made from polyvinyl chloride (PVC) sheets must be cleaned once or twice every two weeks to be more transparent [15]. On the other hand, regular cleaning is crucial to maintain the solar panel efficiency integrated on the greenhouse roof by removing dust [16]. Cleaning greenhouse roofs manually can be time-consuming, dangerous, and cause damage to the roofs themselves. A greenhouse roof-cleaning has been developed to overcome these challenges [17]. These robots use global systems for mobile communication (GSM) technology to ensure that the cleaning process is started and completed reliably. Using a cleaning robot to remove debris from greenhouse roofs can boost plant development when the robot is programmed to receive signals from the operator [18]. The robot cleans automatically, reducing the risk of accidents for human workers.

Greenhouses can be cleaned manually or with technology. Concerning manual cleaning, Mijinyawa and Akpenpuun [19] developed a handheld cleaning device equipped with a rotary brush and water sprayer that can be easily extended and retracted, making it the perfect tool for dust-free greenhouse cleaning. Meanwhile, applying cleaning technologies, Kong et al. [20] designed a multi-span greenhouse cleaning machine with a brush to remove dust and a cleaning brush to wash away dirt and sludge. It has three main parts: walking, cleaning, control, and a mobile platform for automatic shifting and precise control. In the same vein, Nie et al. [21] developed a cleaning device specifically for greenhouse film roofs, showcasing its remarkable attributes, including high cleaning efficacy and efficiency, notable water-saving capabilities, superior safety features, and lightweight design. Patil et al. [22] developed a semi-automatic roof-cleaning device capable of effectively removing dirt, grime, algae, and other substances without causing any harm to the structure or cover of the greenhouse. Similarly, He et al. [23] designed a machine for cleaning plastic greenhouses that improves light transmission by determining the number of rubs and pulls per unit length. In addition, Çabuk [24] designed a four-legged glass cleaning robot specifically for Venlo-style greenhouses, but unfortunately, it has a low efficiency level. Furthermore, Tianhua et al. [25] developed a cleaning machine that fully meets Venlo-type greenhouse roofs' cleaning requirements. Furthermore, Xu et al. [26] developed a greenhouse roof cleaning system utilizing Adams dynamic simulation software to enhance the stability and dependability of the cleaner across diverse working conditions. Amin et al. [27] developed a solar-powered robotic system to address the issue of dust accumulation on multi-span greenhouse roofs, which significantly affects light transmission and plant growth. This system was designed to automate the cleaning process in large-scale greenhouse operations, thereby increasing light intensity and improving energy efficiency.

Hence, it is crucial to find a proper solution to the problem of dirt accumulation and water condensation on the plastic cover of greenhouses. Therefore, this study aimed to develop a cleaning device for greenhouse covers to be more efficient and provide more light intensity for plant photosynthesis. The proposed solution provides a practical and affordable option for farmers with limited resources, while contributing to the advancement of efficient greenhouse maintenance technologies. Moreover, it decreases the damage caused by covering materials and saves water and time consumption.

## 2 Materials and methods

This section describes the manufacturing and development of a cleaning device using a mist blower to clean greenhouse roofs in Nanjing City, China.

### 2.1 Description of the cleaning device

The developed greenhouse roof cleaner device comprises four parts: a motorized knapsack sprayer, a cleaning arm, spraying nozzles, and a cleaning brush and wiper.

#### 2.1.1 The motorized knapsack sprayer

The motorized knapsack sprayer (Model KDK-900), features a single-cylinder, 2-stroke, air-cooled engine with a displacement of 25.6 cc and an engine power of 0.9 kW (1.2 hp). It operates on a 25:1 fuel mixture with a 700 ml fuel tank. The sprayer also includes a 30 L tank, an 18 mm plunger diameter, a pressure range of 1.5–2.5 MPa, an output of 8 L/min, a weight of 12 kg, and rotation speeds ranging 2400–6160 rpm (low engine speed – high engine speed). Its pump is constructed from brass, aluminum, and plastic. Anti-vibration mountings were used to affix the engine and fan assembly to the metal frame of the backrest. The sprayer had padded backrests and straps.

#### 2.1.2 The cleaning arm

**Fig. 1:**
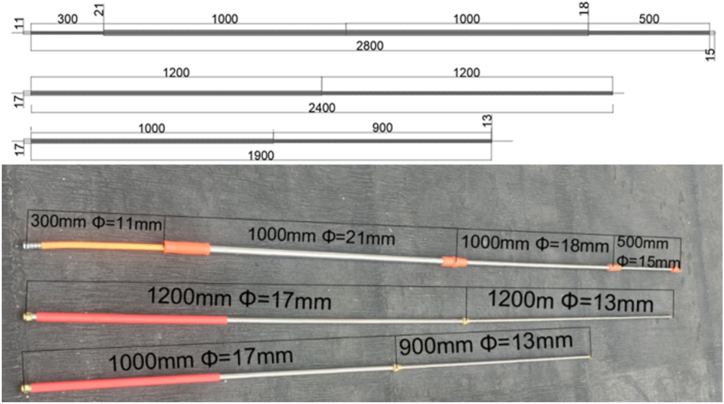
Cleaning (Telescopic) arm.

The cleaning arm has a three-section telescopic mechanism. The cleaning arm comprised three concentric pipes with 21-, 18-, and 15-mm internal diameters. The lengths of these pipes were 1000, 1000, and 500 mm, respectively, as shown in Fig. 1. The pipes were arranged in descending order of diameter, with the largest at the bottom and the smallest at the top. The arm permanently connects the bottom and middle pipes, while the upper pipe can be extended or retracted into both the middle and bottom pipes using a lock mechanism. The cleaning arm was designed to prioritize weight reduction, and subsequent testing assessed its resistance to bending stress and deflection. The handle was fixed in place, whereas the brush end was unrestrained, the brush weight acting as the load.

#### 2.1.3 The spraying nozzles

**Fig. 2:**
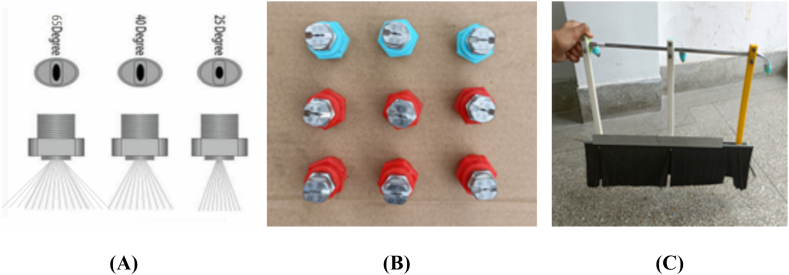
Spraying nozzles with three spray angles. (**A**) Spray angles of 25°, 40°, and 65°, (**B**) Spraying nozzles used in the study, (**C**) Device assembly with attached spraying nozzles.

The spraying nozzles are critical in ensuring the proper water jet spray for effective cleaning. The traditional pure water sprays have poor dust removal effects, and the principles of droplet wetting and dust encapsulation need to be clarified, resulting in inefficient dust removal [28]. So, the appropriate nozzle selection demands careful consideration of various factors, including the cleaning area, operating pressure, and attachment options. For a wider coverage and the ability to withstand 4500 psi, a 40-degree flat spray pattern nozzle is recommended. Multi-nozzle boom spray flat fan nozzles were utilized in three-nozzle steel boom sprayers, with three spray angles of 25°, 40°, and 65°, respectively. These nozzles were grouped together, and the amount of water consumed during the cleaning process was measured in the laboratory and the field. A sprayer handle was designed using a 3D printer to fit the arm and enhance usability. The connection between this process and the nozzles was established using three plastic connections manufactured with a 3D printer, as shown in Fig. 2.

#### 2.1.4 The cleaning unit

**Fig. 3:**
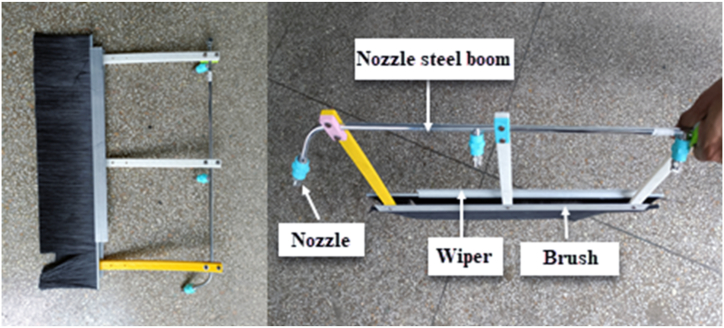
Components and installation of the cleaning unit.

The cleaning unit contains a brush, wiper, and water sprayers (nozzles). The cleaning brush made of synthetic fiber was responsible for sediment removal and has a length of 550 mm, corresponding to the water area covered by three nozzles. The brushes are mounted on a rectangular aluminum frame measuring 305 × 610 mm, using 10 cm bolts and nuts. The brush is directly attached to the upper arm and equipped with three nozzles that evenly spread the cleaning mix. The synthetic fibers efficiently clean dirt through the back-and-forth motion of the operator's arms. The wiper was attached to the brush frame, which was used to wipe the cleaning mixture along the brush length during cleaning. The wiper is made of rubber and measures 40 cm in length, 3 cm in width, and 20 cm in height. It is clamped with sweep angle control. A water reel was also installed on the arm to supply the washing mixture to the brush, as shown in Fig. 3. Construction materials were selected for suitability, availability, and cost-effectiveness, prioritizing affordability for local artisans. Primary materials comprised stainless and galvanized mild steel, aluminum, and fibrous materials widely accessible in various markets. Stainless steel was recommended for the brush attachment to reduce weight and facilitate easy lifting. Three parts were used to install the nozzles on the spray arm, and a 3D printer (Model: i3 MK3, Prusa, Czech) was used to create the mattress and water wiper. The brush was attached to the spray arm using three joints, 20 cm long and 2 cm wide. This setup allows cleaning angles to be adjusted. The entire device was designed using the SolidWorks program version 16.4.

### 2.2 Field tests and operators

The trials were carried out on a single-span greenhouse of arch-detached type, measuring 6 m wide, 40 m long, and 3 m in height. This greenhouse was covered with a single layer of 0.15 mm Polyethylene (PE). The experiments were conducted at the College of Engineering, Nanjing Agricultural University, China, with the experimental site coordinates being a latitude of 32°08′56″N, a longitude of 118°41′68″E, and an altitude of 32 m. The experiments were conducted to clean the greenhouse cover at three heights of 2.8, 2.4, and 1.9 m to enhance the brush handling at different heights.

**Fig. 4:**
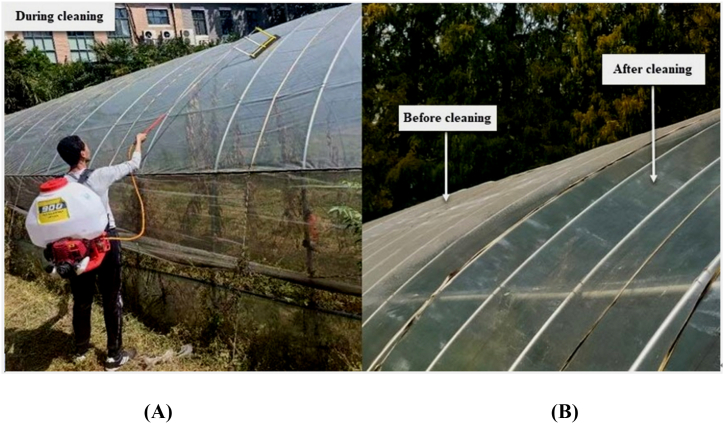
Evaluating the performance of the cleaning device. (**A**) Cleaning device during operation, (**B**) Greenhouse roof before and after cleaning.

Four physically fit men with prior experience working with mist blowers, ranging in age from 21 to 45 years (average 33 years), were carefully selected to evaluate the performance of the developed cleaning device, as shown in Fig. 4. Their body weights ranged from 62 to 90 kg (mean 76 kg). Their heights ranged from 1.71 to 1.85 m (average 1.79 m). Each operator's body mass index (BMI) was computed separately, as shown in Table 1 [29], adhering to the recommended procedures of ISO 2631-1 for evaluating the discomfort induced by mist blower vibrations in varying working conditions and tank fill levels [30].

**Table 1:** Operator's body mass index (BMI).

BMI class	Code	World Health Organization (WHO) definition	Number of operators in each class
21.2	1	Normal range	25 %
23.7	2	Superior normal range	25 %
27.2	3	Overweight	25 %
80.8	4	Pre-obese	25 %

### 2.3 Design calculations and material selection

The selection of materials, design calculations, and thorough mathematical and scientific analysis were done to ensure that the product meets safety and efficiency standards. These factors impact everything from durability and cost-effectiveness to the effect on the worker, greenhouse cover, and innovation, as shown in Table 2.

**Table 2:** Material selection and proprieties.

	Wiper	Brush	Three-nozzle steel boom	Telescopic arm	Knapsack sprayer
Dimensions (mm)	6 × 80	3 × 80		(1) 2800 (2) 2400 (3) 1900	
Wight (kg)	0.13	0.19	0.30	(1) 0.74 (2) 0.33 (3) 0.40	12

#### 2.3.1 Bending moment criterion

Through the locking system, the integrated behavior of the three concentric pipes allows for the calculation of the bending stress in the smallest cross-sectional area as follows (Eqs. (1), (2), (3), (4))):

The bending moment due to the brush was calculated as follows:

(1) $$M_{b}=\frac{wb\times l}{2}$$

(2) $$M_{h}=\frac{wb\times l}{2}$$

(3) $$Z=\frac{\pi \left(D^{3}-d^{3}\right)}{32}$$

(4) $$Bending\;stress=\frac{Maximum\;bending\;moment}{Section\;modulus}$$

where *Mb* is the brush's bending moment, *wb* is the brush's weight, *l* is the handle's length, *M*_*h*_ is the handle's bending moment, *Z* is the section modulus, *D* is the outer pipe diameter, and *d* is the inner pipe diameter.

#### 2.3.2 Deflection

One can determine the maximum deflection by considering the arm as a singular beam with a defined length and utilizing the minimal cross-sectional area. This deflection is the combined result of both the arm's self-weight and the supported brush. The calculation for this deflection is as follows (Eqs. (5), (6))):

(5) $$\Delta 1=\frac{wb\times l^{3}}{3EI}+\frac{wh\times l^{3}}{3EI}=\frac{\left(wb+wh\right)}{3EI}\times l^{3}$$

(6) $$I=\frac{\pi \left(D^{4}-d^{4}\right)}{64}$$

where *Δ1* stands for the maximum deflection, *E* stands for Young's modulus, *I* represent inertia moment, and *l* stands for the handle length. The permissible deflection was given as 0.0031 mm. Consequently, the deflections for the three arms were calculated as 8.4 mm (0.003 × 2800), 7.2 mm (0.003 × 2400), and 5.7 mm (0.003 × 1900), respectively. All the calculated deflections exceeded the maximum allowed, ensuring the safety of the arms.

### 2.4 The hydrodynamic behaviors of fluid in the cleaning section

This section focuses on fluid dynamics and computational fluid dynamics (CFD) applications to analyze fluid motion. ANSYS Fluent is a computational tool that examines fluid flow behavior within the cleaning section of the system, from the inlet to the outlet. The ANSYS Fluent software can provide precise predictions using CFD techniques to anticipate fluid flow and related phenomena [31].

#### 2.4.1 Computational fluid dynamics using ANSYS fluent

The finite element approach, a promising method for fluid flow study with CFD techniques, involves dividing the body into small elements. Analyzing each particle in the body is a challenging task that meshing addresses by breaking down the body into smaller elements. Meshing discretizes the domain into smaller volumes for iterative solving, beginning with setting conditions in the domain and leading to modeling the entire system; this process was used to analyze the results in this study.

#### 2.4.2 Geometry and mesh

**Fig. 5:**
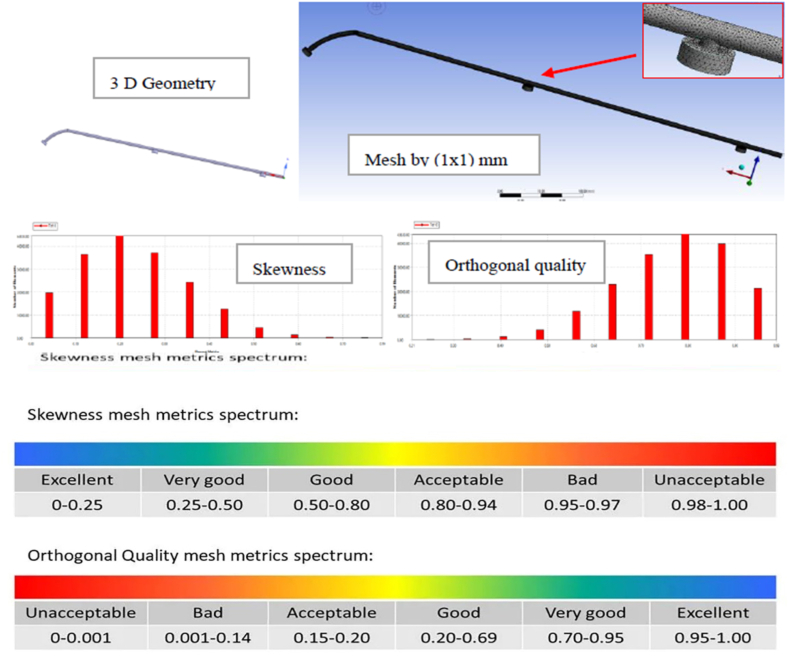
The spectrums of skewness and orthogonal quality mesh metrics and the representation of 3D geometry and mesh of a pipe model.

The experiment's geometry was created using ANSYS Fluent® version 2023 R2 and the Workbench Design Modular tool, which underwent a computational fluid dynamics (CFD) analysis to determine pressure drops and velocity within the pipe. A three-dimensional model, reflecting the actual dimensions of the pipe, was used and enhanced precision compared to a two-dimensional model [32]. The default mesh was improved by integrating supplementary controls to optimize mesh quality, focusing on elements, skewness, and orthogonal quality. Various meshing techniques, including proximity and curvature functions, were applied to achieve a mesh quality of at least 0.8. The "patch conforming" mesher utilizing "tetrahedrons" emerged as the preferred approach, demonstrating superior accuracy, especially in capturing curvatures, among multi-zone, hexagonal dominant, and sweep methods. Fig. 5 displays standard skewness and orthogonal quality mesh metrics spectrums.

**Fig. 6:**
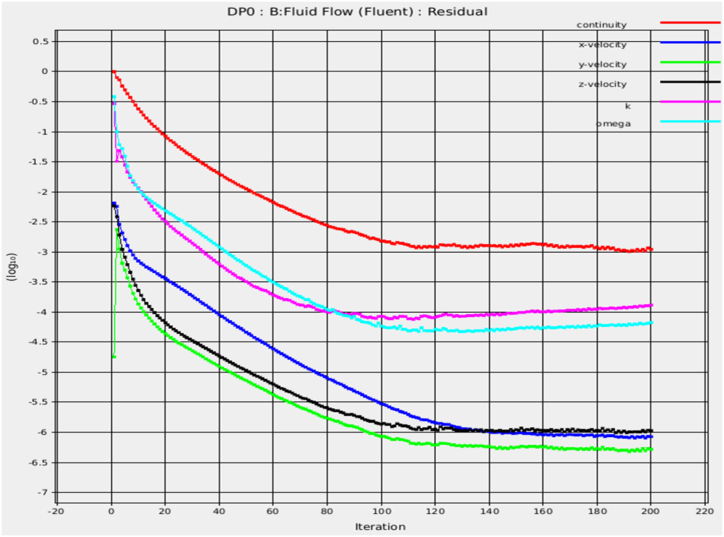
Convergence solution.

Fig. 5 illustrates the geometry and mesh of the cleaning section used in this study. The geometry had a tube length of 590 mm and an inner pipe diameter of 6 mm. It was meshed into finite elements at a size of 1 mm × 1 mm, with inlet, outlet, and wall boundary conditions. The cleaning section model was meshed using 274454 nodes and 178750 elements, as shown in Fig. 6. The meshing method achieved an average element quality of 0.81, an average skewness quality of 0.22, and an average orthogonal quality of 0.85, confirming the high quality of the meshing used in this study. These results agree with Fatchurrohman and Chia [33]. The physical properties of the used water were include a density of 1000 kg/m³, thermal conductivity of 0.606 W/m.K, dynamic viscosity of 300 kg/m.s, velocity of 0.001002 m/s, Reynolds number of 0.248, and a flow type characterized as turbulent flow with a corresponding value of 148109.

#### 2.4.3 Validation based on laminar and turbulent

ANSYS Fluent is a popular CFD software tool with a long-standing reputation for simulating fluid flow in pipelines. The study aimed to simulate and analyze the fluid flow in a pipe, considering pressure drop and velocity profile. This was done to validate the developed methodology. This study examined how different pressures affect the velocity of water flowing through a pipe. The physical properties of the fluid and its initial values were used as input parameters. The simulation was conducted using ANSYS Workbench, which generated 3D structures representing the pipe's geometry.

Further simulation was conducted using a solver using the steady-state fluid flow approach. The turbulent flow in the pipe was modeled using a standard k-model, where 'k' represents turbulent kinetic energy, and 'ε' denotes the dissipation rate. Alternatively, turbulence models aligned with mean flow equations can be considered. The study also investigated Reynolds-averaged Navier-Stokes (RANS) models. In this study, a standard k-model was favored for turbulent flow. The governing equations of motion, known as Navier-Stokes equations in cylindrical coordinates, apply to incompressible Newtonian fluids and include mass conservation (Eqs. (7), (8))).

(7) $$\frac{\partial \rho }{\partial t}+\frac{1}{r}\frac{\partial }{\partial r}\left(\rho rv_{r}\right)+\frac{1}{r}\frac{\partial }{\partial \theta }\left(\rho v_{\theta }\right)+\frac{\partial }{\partial z}\left(\rho v_{z}\right)=0$$

Momentum conservation:

(8) $$\rho \left(\frac{\partial v}{\partial t}+v_{r}\frac{\partial v_{r}}{\partial r}+\frac{v_{\theta }}{r}\frac{\partial v_{\theta }}{\partial \theta }+v_{z}\frac{\partial v_{z}}{\partial z}\right)=-\frac{\partial \rho }{\partial z}+\rho g+\mu \left[\frac{\partial }{r\partial \theta }\left(r\frac{\partial v_{r}}{\partial r}\right)+\frac{\partial^{2}v_{\theta }}{r^{2}\partial \theta^{2}}+\frac{\partial^{2}v_{z}}{\partial z^{2}}\right]$$

At steady-state flow, velocity has only an axial component expressed as a function of radial coordinates governed by balancing weight, pressure drop, and viscous forces.

#### 2.4.4 Transient fluid flow through the pipe

Transient flow is a type of fluid flow that is time-dependent and usually unsteady. ANSYS Fluent is a software that can study this type of flow. When the fluid has high viscosity, it flows along the outlet as a well-defined line due to the molecular diffusion of the fluid. However, the flow varies with time for fluids with lower viscosity, creating irregular bursts due to unbalanced behavior appearing along the streak [32]. The fluid with lower viscosity streaks immediately and spreads randomly across the entire pipe, producing a blurred effect.

### 2.5 Measurements

#### 2.5.1 Light measurement

The light intensity was measured hourly throughout daylight using a luxmeter (Model TA632 A/B) manufactured in China. The Luxmeter has a measuring range of (0.1–200,000 lux) and an accuracy of ± (3 % + 5 lux). Light intensity was measured at three distinct internal and external locations within the greenhouse structure at five different elevations (A = 2.5 m, B = 2.0 m, C = 1.5 m, D = 1.0 m, and E = 0.5 m) from the ground. All measurements were obtained following the complete desiccation of the roof. The experiments were conducted in December and October 2023 during the day from 8 a.m. to 5 p.m.

The primary objective of the cleaning procedure was to improve the plastic material's light transmittance. A comparative analysis of the plastic material's transmittance before and after cleaning was performed by Zhou et al. [34], as follows in using Eqs. (9), (10)).

(9) $$T_{\lambda 1}=\frac{I_{1}}{I_{o}}$$

(10) $$T_{\lambda 2}=\frac{I_{2}}{I_{o}}$$

where $T_{\lambda 1}$ is transmittance before cleaning (%), $T_{\lambda 2}$ is transmittance after cleaning (%), *I*_*o*_ is the incident light illumination (lux), *I*_*1*_ is the transmitted light illumination before cleaning (lux), and *I*_*2*_ is the transmitted light illumination after cleaning (lux).

Consequently, the cleaner's efficacy was evaluated by comparing pre-and post-cleaning light transmission values through the roof.

#### 2.5.2 Fuel, water and time consumption

The fuel consumption rate for the gasoline-powered machine was determined by using Eq. (11), as follows below:

(11) $$FC=\frac{f}{t}\times 3.6$$

where *FC* is the fuel consumption rate (l/h), *f* stands for the volume of consumed fuel (cm^3^), and *t* is the operating time (s).

A calculation of the water consumption rate during the cleaning procedure was performed using Eq. (12), as follows below:

(12) $$WC=\frac{w}{t}$$

Here, *WC* denotes the water consumption rate (l/h), *w* represents the volume of water consumed water (l), and *t* signifies the operating time (h).

#### 2.5.3 Cleaning efficiency and performance rates

All cleaning device parts were correctly connected, and the knapsack sprayer tank was filled with water to check the connections before starting the initial operation. A top-down cleaning approach was implemented, ensuring all greenhouse sections were thoroughly cleaned; meanwhile, the telescopic joint was used to control the heights and cleaning process.

The rate of theoretical productivity (GH/h) was determined using Eq. (13):

(13) Pt=S×W

Here, *Pt* represents the theoretical productivity, *S* signifies the forward operator speed, and *W* denotes the swath width.

The actual cleaning time was determined using Eq. (14):

(14) $$\text{Actual}\;\text{time}=t_{1}+t_{2}+t_{3}+t_{4}$$

Here, *t*_*1*_ represents the spraying time, *t*_*2*_ denotes the turning time, *t*_*3*_ signifies the fill-up tank time, and *t*_*4*_ implies the over-lapping time.

Field efficiency was calculated according to the method presented in Eq. (15):

(15) Fieldefficiency=Theoreticaltime×Actualtime×100

Eq. (16) calculates the rate of actual productivity (GH/h) as follows:

(16) Pa=Pt×η

Here, *Pa* denotes the actual productivity, *Pt* represents the theoretical productivity, and *η* signifies the field efficiency.

The performance rate was calculated according to the method presented in Eq. (17):

(17) $$\text{Performance}\;\text{rate}=\text{Pa}\times h\;\left(m^{2}/\text{day}\right)$$

Here, *h* represents the daily work hours (assumed to be h = 6 h).

## 3 Results and discussion

The findings indicated that the mean light intensity before cleaning was 25311.2 lux, whereas the post-cleaning value was 35539.4 lux, with an external light intensity of 51931 lux during the experiment. As a result, the light transmittance of the greenhouse roof increased from 48.7 % to 68.5 % after cleaning. A significant increase of 19.8 % in greenhouse illumination is expected to improve crop production significantly. These results are consistent with Zhou et al. ]34[ who reported that optimal light intensity at varying temperatures has improved the plant's photosynthetic capacity and yield.

### 3.1 CFD (ANSYS Fluent) results

The generated results by the ANSYS Fluent program were visually represented in 3D formats. The numerical solution obtained from the Ansys simulation is illustrated in Fig. 6. The displayed diagram demonstrates the attainment of stability through the iterative process, implying the successful convergence of the simulation. This convergence proves the simulation's ability to generate consistent and reliable outcomes, validating its accurate system representation within the defined parameters. The convergence criterion plays a crucial role in assessing the dependability of the numerical solution, affirming its appropriateness for deriving meaningful conclusions within the scope of this research.

To ensure the findings' credibility, extensive validation of the numerical simulations was conducted. The ANSYS Fluent program was used to create detailed 3D visualizations (Fig. 6) to represent the simulation results. However, the validation did not stop there. Thorough validation procedures were performed to confirm that the simulations were consistent and reliable within specific parameters. This rigorous validation demonstrates the strength of the numerical solutions and confirms their suitability for providing valuable insights aligned with the research goals.

**Fig. 7:**
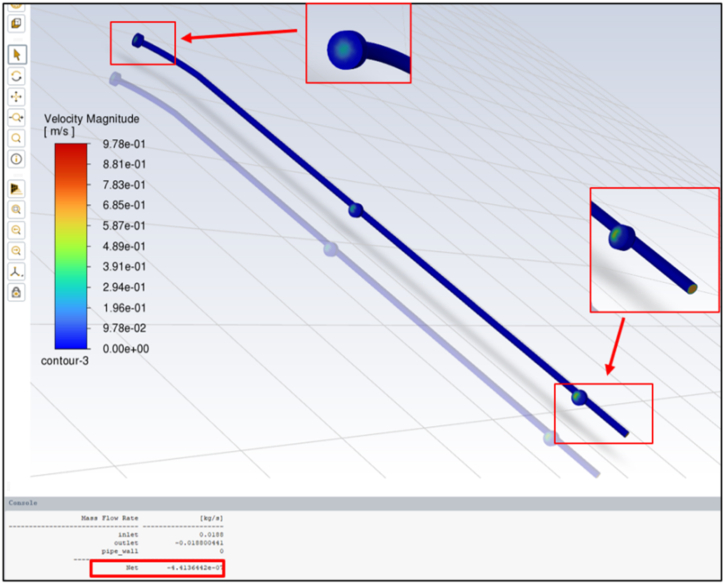
The net mass flow rate and the velocity vector of water flow in the cleaning section.

The simulation's net mass flow rate is nearly zero, approximately −4.41 × 10^-7 kg/s, as indicated in Fig. 7. This result demonstrates well-balanced inflow and outflow rates, which are crucial for maintaining effective mass conservation and ensuring system stability. In addition, this low value could confirm the robustness of the simulation, highlighting minimal mass imbalance and accurate fluid flow dynamics, which are essential for the consistent performance of the greenhouse roof cleaning process [35,36].

The velocity distribution across the nozzles revealed that the water velocity at the entry point was the highest, followed by the next nozzle to the entry hole, and the lowest water velocity was at the last nozzle. However, there was no significant effect on the consistency of the water flow rates from the three nozzles.

**Fig. 8:**
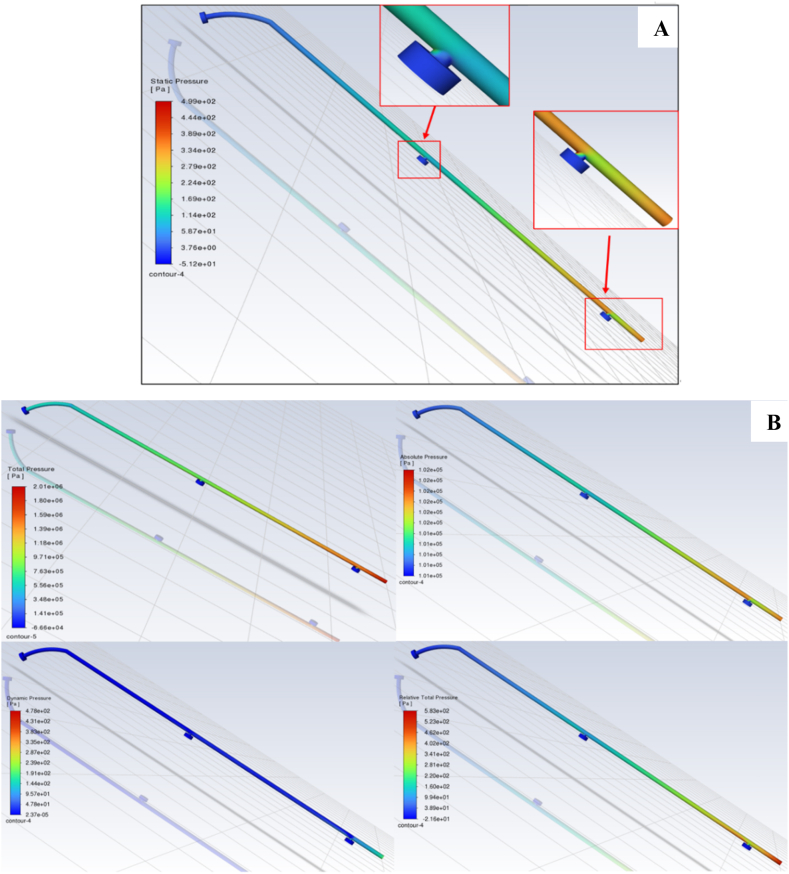
The static pressure of water flow in the cleaning section **(A)** and the pressure parameters of water flow in the cleaning section (**B)**.

Fig. 8A shows the results of a static pressure analysis of the cleaning pipe, which reveals a meticulously controlled pressure distribution along the cleaning arm. The first nozzle close to the inlet showed a higher pressure than the subsequent nozzles. The static pressure gradually decreases as the water progresses along the cleaning arm. Thus, the last nozzle showed a lower pressure than its counterparts. This balanced pressure profile is essential for achieving a uniform water flow across all nozzles and maintaining a consistent cleaning performance [37,38].

Additionally, Fig. 8B analyzes key pressure parameters, including dynamic pressure, absolute pressure, total pressure, and stagnation pressure. These findings provide critical insights into the design's precision and effectiveness. Moreover, it could show its capability to meet safety, accuracy, and performance objectives in an automated greenhouse cleaning system [39,40].

**Fig. 9:**
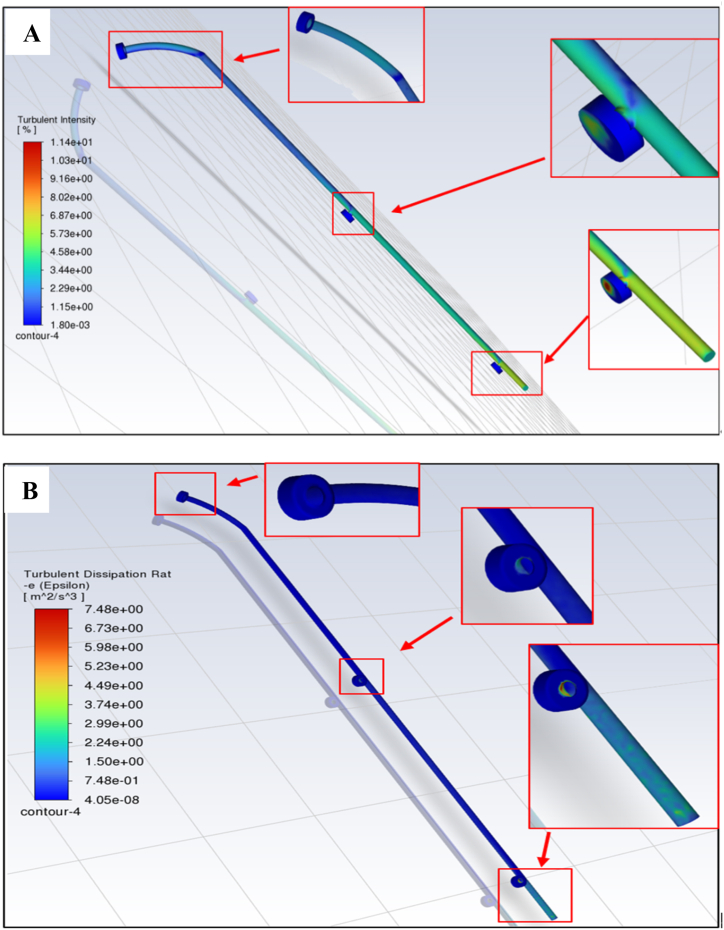

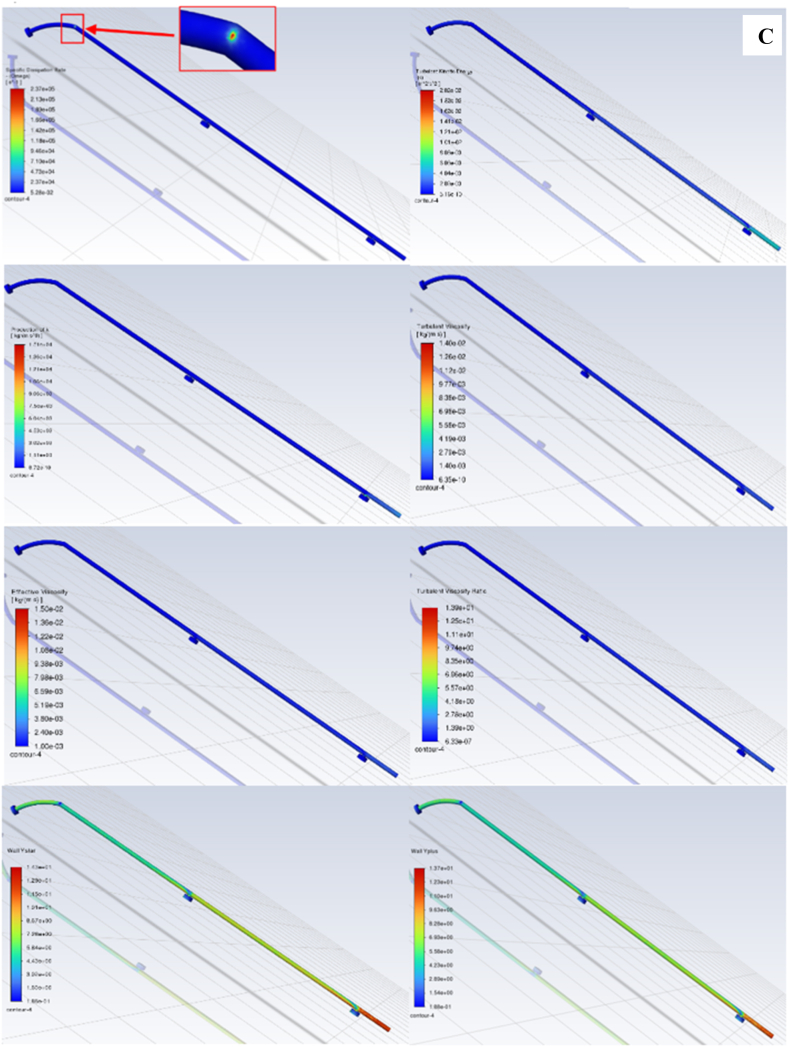
The turbulent intensity of water flow in the cleaning section **(A)**, the turbulent dissipation rates **(B),** and exploring turbulent dynamics: key factors influencing the cleaning section pipe performance **(C)**.

Fig. 9A provides an in-depth analysis of turbulent flow intensity. This analysis could accurately assess water velocity fluctuations and turbulent levels, confirming that the design is effective and emphasizing the importance of turbulence control in ensuring stability and efficiency. Additionally, the study provides valuable predictions of heat transfer rates, a crucial factor that influences the entire system's performance, stability, and efficiency. This comprehensive understanding derived from turbulent intensity analysis could enhance the design approach's credibility and has significant implications for optimizing the system's thermal and operational characteristics.

The study has delivered positive outcomes from the turbulent dissipation rate, which signifies significant progress. These results enhance the capability of the turbulence models and highlight their effectiveness in accurately depicting the complexities of turbulent flows (Fig. 9B**).**

Fig. 9C illustrates the impact of various factors such as turbulent kinetic energy, specific dissipation rate, k-yield, turbulent viscosity, effective viscosity, turbulent viscosity ratio, wall Y Star, and wall Y Plus on water dynamics within the cleaning arm. The study provides valuable insights that can optimize design efficiency, understand turbulence effects, and improve predictive modeling to enhance system performance and reliability. Additionally, the research contributes to advancements in heat transfer mechanisms, mitigation of pressure loss, and ensuring the structural integrity of the cleaning system.

**Fig. 10:**
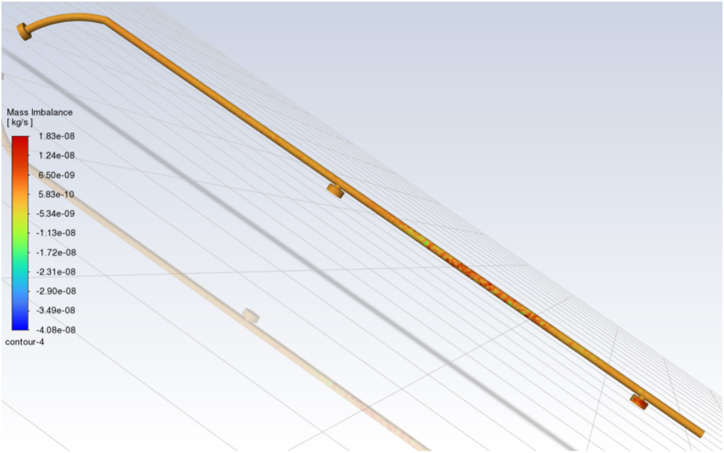
The mass imbalance in the cleaning section: Improving efficiency, conservation, and system performance.

The analysis of the mass imbalance in the cleaning section, as shown in Fig. 10, has revealed that the system was free from leaks or breaches and maintained optimal water levels. The study highlights the efficiency of water distribution systems, ensuring a smooth flow to sprinkler nozzles without any avoidable losses. The results indicate consistent water pressure and regular flow rates, which contribute to the overall performance of the cleaning system.

### 3.2 Bending moment and deflection effect

The design and assembly of the telescopic arm were critical for ensuring the greenhouse roof cleaning device's structural integrity and operational efficiency. The arm consists of three concentric tubes that operate in unison, facilitated by a precision-engineered locking system. This system ensures that the tubes maintain their alignment, allowing them to extend and retract smoothly while bearing the load of the cleaning brush.

**Fig. 11:**
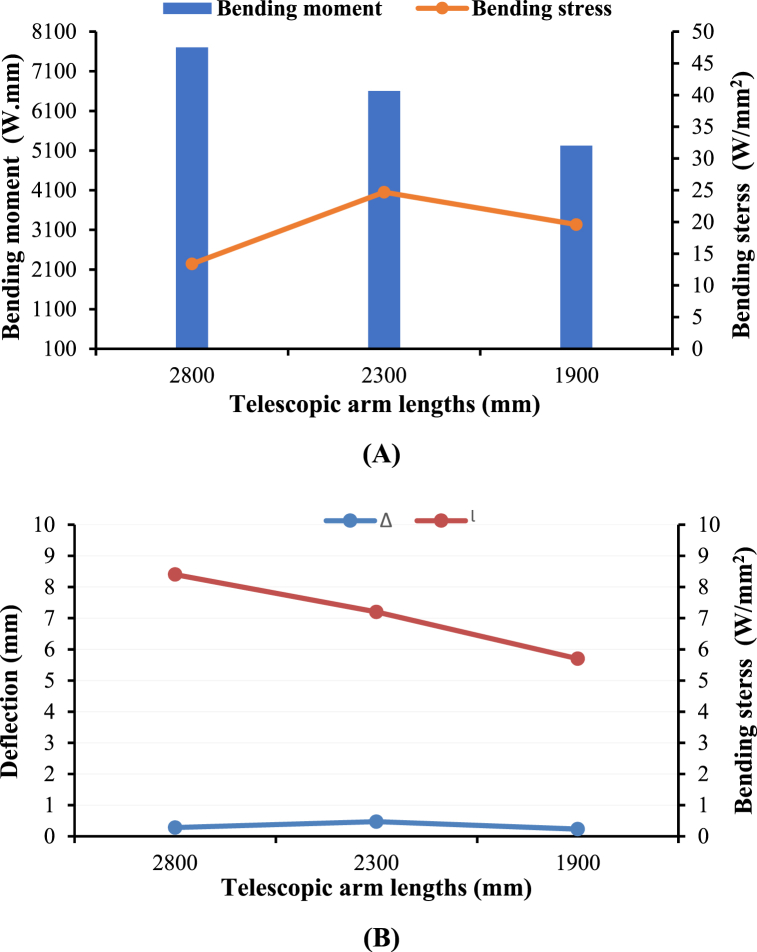
The relationship between bending moment and bending stress for three lengths of telescopic arm **(A)** and between deflection and bending stress for three lengths of telescopic arm (**B)** (Note: Δ the maximum deflection, and *l* the handle length).

The telescopic arm functions as a cantilever, with the fixed end attached to the device and the free end supporting the cleaning brush, wiper, and nozzles. The bending stresses experienced by the arm during operation were analyzed at three different extended lengths: 2800 mm, 2300 mm, and 1900 mm. These lengths were chosen to represent the arm's typical operational range. The corresponding bending stresses were calculated to be 13.4 W/mm^2^, 24.7 W/mm^2^, and 19.6 W/mm^2^, respectively, as shown in Fig. 11A [41,42].

These stress values are significantly lower than the yield strength of mild steel, which was 500 N/mm^2^ [43]. This margin of safety ensures that the arm will not experience plastic deformation or failure during regular operation. The lower stress values also indicated that the arm can safely carry the cleaning brush, wiper, and nozzles without compromising its structural integrity, even at the maximum extension.

Further analysis was conducted to assess the deflection of the telescopic arm under load. Using Eqs. (5), (6)), the allowable deflection was calculated with an allowable deviation of 0.003. The deflections result for L1 (2800 mm), L2 (2300 mm), and L3 (1900 mm) were 8.4 mm, 7.2 mm, and 5.7 mm, respectively. These values exceed the maximum calculated deflection. Thus, the arm could maintain rigidity and stability even under significant load. As shown in Fig. 11B, the locking system effectively prevents excessive deflection, ensuring the arm remains secure during operation. This analysis is consistent with the stress analysis principles outlined by Davis [44] for high-performance mechanical components, ensuring the arm was secure in both telescopic connections.

The low bending stress and minimal deflection combination confirm that the telescopic arm design meets safe and effective operation criteria. The arm's ability to extend and retract while maintaining structural integrity is vital for the device's performance, especially when cleaning more extensive or complex greenhouse roofs.

### 3.3 Influence of motor speed and nozzle angle on the water flow rate through nozzles

**Fig. 12:**
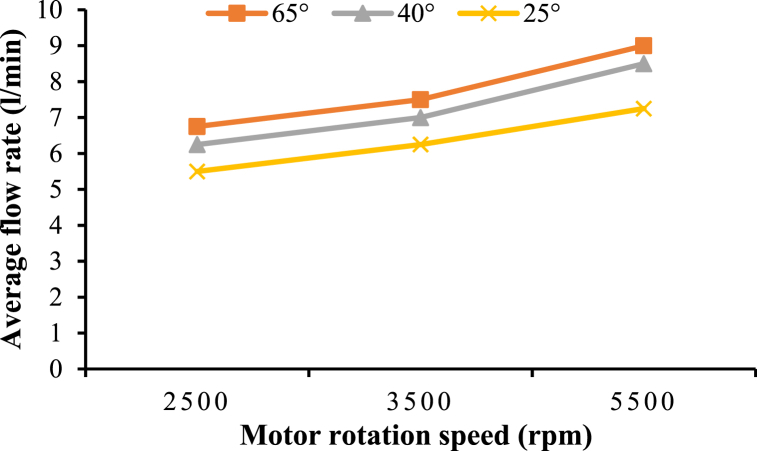
Influence of the motor's rotational speed and nozzle angle on the nozzle's flow rate.

The motor rotational speed is crucial in determining the discharge of nozzles for cleaning the greenhouse roof. As illustrated in Fig. 12, increasing the motor speed results in a proportional increase in the flow rate across all tested nozzle angles [45,46]. This relationship is critical for optimizing the device's cleaning efficiency, as the flow rate directly impacts the water used for cleaning the roof surface.

The experiments showed that increasing the motor speed from 2500 rpm to 5000 rpm increased the water flow rate by 24.14 %, 26.47 %, and 25.00 % at nozzle angles of 25°, 40°, and 65°, respectively. These findings align with previous research conducted by Bateman [47], who reported similar trends in the nozzle flow rate as a function of motor speed.

The influence of the nozzle angle on the flow rate was also examined. Results indicated that the larger the nozzle angles, the higher the flow rate. Therefore, increasing the nozzle angle from 25° to 65° resulted in an increase of flow rate from 5.50 to 6.75 l/min, 6.25–7.50 l/min, and 7.25–9.00 l/min at motor speeds of 2500 rpm, 3500 rpm, and 5500 rpm, respectively. This trend can be attributed to the broader spray pattern produced by larger nozzle angles, which allow more fluid to be dispersed over a wider area.

The ability to adjust motor speed and nozzle angle provides the flexibility to tailor the cleaning process to specific conditions, such as varying levels of dirt accumulation or different roof geometries. By optimizing these parameters, the device can deliver consistent and effective cleaning performance, regardless of the operating environment.

### 3.4 Effect of motor speed on nozzle angle in boom sprayer

**Fig. 13:**
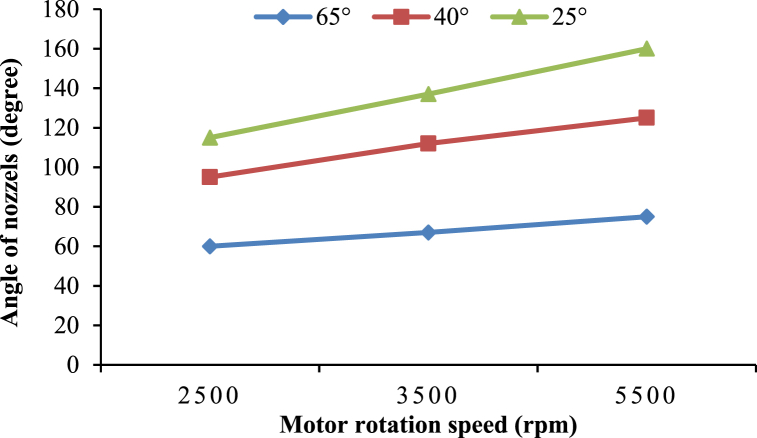
Relationship between the average of nozzles and rotation speed.

Fig. 13 shows that increasing the motor speed increases the flat fan nozzle angle in the boom sprayer [48]. Therefore, the angle of the sprayer cone was ranged from 25° to 35° at 2500 rpm to 65°–75° at 5500 rpm. This expansion in the nozzle angle is beneficial for achieving greater coverage during the cleaning process, particularly on larger roof areas where uniform application of the cleaning fluid is essential.

### 3.5 Practical implications and design optimization

The design of the greenhouse roof cleaning device relies heavily on the telescopic arm and motor rotation speed. The telescopic arm's capacity to extend and retract while maintaining structural integrity enables the device to navigate different roof configurations efficiently. This adaptability is crucial for greenhouses' large roofs and structures, allowing the arm to reach distant areas without compromising stability.

The low bending stresses and minimal deflection in the telescopic arm design ensure the device can operate safely even under heavy load conditions. This reliability is crucial for maintaining the system's longevity and preventing mechanical failures during the operation. The device design also allows for the potential scaling of the system to be used in larger greenhouses or commercial applications where durability and consistency are paramount.

In parallel, the motor rotation speed directly influences the cleaning efficiency by controlling the nozzles' flow rate and spray angle. Adjusting these parameters enables the device to adapt to different cleaning scenarios, such as varying dust and dirt buildup levels. For instance, the high motor speeds and wide nozzle angles can be used for quick and high-coverage cleaning. In contrast, low speeds and narrow angles might be more appropriate for cleaning the stubborn dirt spots.

Integrating these features into the design could enhance its operational efficiency and ensure that it can meet the diverse needs of different greenhouse environments. By optimizing the telescopic arm length and motor rotation speed, the cleaning device could provide a flexible and effective solution for maintaining the cleanliness of greenhouse roofs, which is essential for maintaining optimal light transmission and plant growth.

### *3.6* Effect of nozzle height on spray overlapping

Table 3 demonstrates the spray overlapping during the inter-nozzle test at three different angles 25°, 40°, and 65°. The results showed no interference when the mattress arm height was set between 15 and 19 cm, with a nozzle-to-arm distance of 27 cm. The nozzles at the maximum height of 21.5 cm were varied in size from 3 to 5 cm and created an average overlap of only 4 cm at a nozzle angle of 65-degree. Moreover, the identified nozzle overlap varied within the 0–0.05 % range. This reveals a minimal distinction in the overlap between consecutive adjacent nozzles. However, overlapping occurred when the height exceeded 21.5 cm above the mattress arm. The coefficient of percentage for the nozzle at a height of 21.5 cm was considered to be 0.05, while at a height of 17.5 cm, it was merely 0.

**Table 3:** Effect of nozzle height on the overlapping of spray**.**

Overlapping			
25^o^			
40^o^			
65^o^			

### 3.7 Clean efficiency and light intensity

**Fig. 14:**
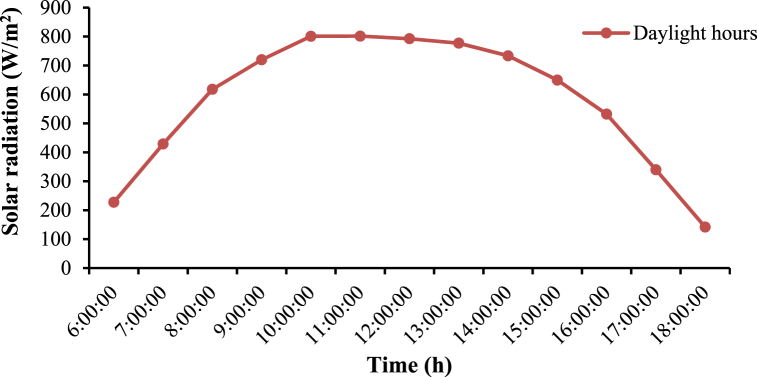
The average solar radiation during the daytime.

The device's efficiency rate was 73.5 %, and it could clean 4.5 greenhouses with a total area of 240 m^2^ for each within a 6-h working day. The light intensity outside the greenhouse was measured on a sunny day by a lux meter and then converted to W/m^2^, as shown in Fig. 14. Fig. 14 shows the solar radiation on a sunny day. The maximum solar radiation on the experimental site was around 800 W/m^2^ at 13:00 and then gradually decreased to 350 W/m^2^ at 17:00.

**Fig. 15:**
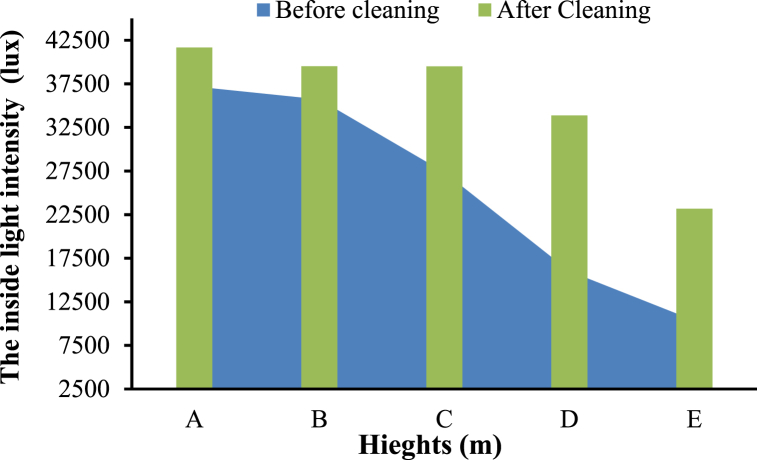
The light intensity before and after cleaning the greenhouse roof.

On the other hand, Fig. 15 shows the results of measuring the light intensity before and after cleaning the accumulated dirt on greenhouse covers. Results found that the highest light intensity value inside the cleaned plastic greenhouse was 41669 lux, and 37189 lux inside the uncleaned greenhouse at the same height of 2.5 m from the ground. The average light intensity at five heights of (A = 2.5 m, B = 2.0 m, C = 1.5 m, D = 1.0 m, and E = 0.5 m) before cleaning were (37189, 35705, 27335, 15998, and 10329 lux) respectively, so the average light intensity was 25311 lux (48.7 %) before cleaning. In contrast, the average light intensity after cleaning were (41669, 39513, 39489, 33870, and 15156 lux) respectively; the average light intensity was 35539 lux, which accounts for 68.5 % of the total light intensity.

The minimum light intensity recorded inside the greenhouse after cleaning was 15156 lux, while it was stood 10329 lux before cleaning at the same height from the ground of 0.5 m. It was observed that the light intensity inside greenhouses decreases as the density of dust on the greenhouse roofs increases. The decrease in light transmittance could range from 9 % to 17.5 % for greenhouse surfaces containing light dust, and this decrease became notably pronounced as the dust density increased. The accumulated dust on a surface can decrease the transfer coefficient by as much as 25 %. The equation for determining transmission reduction depends on the density of dust accumulation rather than passaging time and applies across different weather and regions [48]. Therefore, it can be recommended that the greenhouse close to tall trees be regularly cleaned because of the accumulation of dust and bird droppings. Depending on surface cleanliness, these results could significantly vary in light intensity within different greenhouses. Additionally, it was noted that rainfall within the greenhouse hindered the dust removal process. Consequently, the cleaning machine is recommended to be used directly after rainfall to eradicate clinging dust and diminish the adherence of airborne particles and the propagation of microorganisms.

### 3.8 Water and fuel consumption

**Fig. 16:**
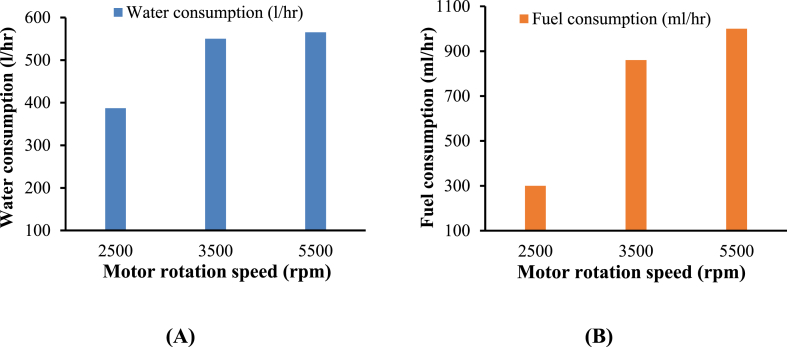
Effect of motor rotation speed on water consumption (**A**) and fuel consumption (**B**) as a function of motor rotation speed.

Fig. 16 presents the effect of the three different motor rotational speeds (2500, 3500, and 5500 rpm) on water and fuel consumption. Results showed that increasing the rotational speed could increase water and fuel consumption. The results also revealed that the highest water and fuel consumption values were 565 l/h and 1000 ml/h recorded at 5500 rpm, respectively. Conversely, the lowest water and fuel consumptions were 387 l/h and 300 ml/h recorded at 2500 rpm, respectively.

Therefore, operating at a high speed of 5500 rpm could increase fuel and water consumption, the rate of vibration and noise, and the percentage of interference between the nozzles. In contrast, operating at a low-speed of 2500 rpm resulted in a decrease in fuel consumption rate by 72 % and a decrease in water consumption by 53 %. However, it also means a low interference ratio and cleaning efficiency.

Accordingly, it is recommended to use a speed of 2500 rpm when dealing with minimal layers of dust. Meanwhile, operating the motor at a speed of 3500 rpm is better in case of high dust accumulation. Adopting this speed makes it possible to achieve superior cleaning performance while ensuring optimal vibrations and noise levels. Meanwhile, the water and fuel consumption decrease, as shown in Fig. 16.

## 4 Conclusion

This study aimed to provide a practical approach to cleaning greenhouse roofs by designing and fabricating cleaning equipment for dust accumulation. Results showed that cleaning the plastic cover of the greenhouse increased its light transmittance from 48.7 % to 68.5 %. Consequently, increased the internal greenhouse light intensity by 19.8 %.

The simulation results of CFD software showed a uniform water flow rate and a controlled pressure distribution of the cleaning arm. Moreover, it was observed that the optimal motor speed was 3500 rpm for optimal cleaning efficiency. The developed device showed several advantages, such as quick operation, low labor demand, low load on the roof, and enhanced labor safety. The fabricated cleaning equipment can fit different greenhouse designs and is easy to operate and install with a low maintenance cost, making it a viable alternative.

Results revealed that operating the motor at a high speed of 5500 rpm changed the nozzle interference, increased fuel and water consumption, and caused noise and vibration. Operating at a low-speed of 2500 rpm decreased fuel consumption by 72 % and water consumption by 53 %; however, it decreased cleaning efficiency. Subsequently, it can be recommended that using the speed of 3500 rpm could achieve a high cleaning efficiency without increasing the water and fuel consumption.

The developed device possesses considerable potential as a viable alternative because of its faster operation, low labor demand, low roof load, and more safety benefits. The developed cleaner can be easily adjusted to fit different gabled-designed and standalone agricultural greenhouses. Therefore, this device can offer a reliable and durable solution for cleaning agricultural greenhouse roofs.

The current cleaning device can be further improved by incorporating a photovoltaic panel as a sustainable energy source. In addition, integrating artificial intelligence into the device could ensure ease of use and provide more comfort and safety for the operator in various aspects.

## CRediT authorship contribution statement

**Ahmed Amin:** Writing – original draft, Visualization, Methodology, Investigation, Formal analysis, Conceptualization. **Xiaochan Wang:** Writing – review & editing, Supervision, Methodology, Funding acquisition, Conceptualization. **Zhao Lianyuan:** Software, Resources, Data curation. **Yinyan Shi:** Writing – review & editing, Investigation, Conceptualization. **Ren Xiaoyan:** Visualization, Formal analysis. **Mahmoud Okasha:** Writing – review & editing, Visualization, Investigation. **Reda Hassanien Emam Hassanien:** Writing – review & editing, Supervision, Conceptualization.

## Data availability statement

Data will be made available on request.

## Additional information

No additional information is available for this paper.

## Declaration of competing interest

The authors declare that they have no known competing financial interests or personal relationships that could have appeared to influence the work reported in this paper.
